# GWAS of Folate Metabolism With Gene–environment Interaction Analysis Revealed the Possible Role of Lifestyles in the Control of Blood Folate Metabolites in Japanese: The J-MICC Study

**DOI:** 10.2188/jea.JE20220341

**Published:** 2024-05-05

**Authors:** Mineko Tsukamoto, Asahi Hishida, Takashi Tamura, Mako Nagayoshi, Rieko Okada, Yoko Kubo, Yasufumi Kato, Nobuyuki Hamajima, Yuichiro Nishida, Chisato Shimanoe, Rie Ibusuki, Kenichi Shibuya, Naoyuki Takashima, Yasuyuki Nakamura, Miho Kusakabe, Yohko Nakamura, Yuriko N. Koyanagi, Isao Oze, Takeshi Nishiyama, Sadao Suzuki, Isao Watanabe, Daisuke Matsui, Jun Otonari, Hiroaki Ikezaki, Sakurako Katsuura-Kamano, Kokichi Arisawa, Kiyonori Kuriki, Masahiro Nakatochi, Yukihide Momozawa, Kenji Takeuchi, Kenji Wakai, Keitaro Matsuo

**Affiliations:** 1 Department of Preventive Medicine, Nagoya University Graduate School of Medicine, Nagoya, Japan; 2 Department of Healthcare Administration, Nagoya University Graduate School of Medicine, Nagoya, Japan; 3 Department of Preventive Medicine, Faculty of Medicine, Saga University, Saga, Japan; 4 Department of Pharmacy, Saga University Hospital, Saga, Japan; 5 Department of International Island and Community Medicine, Kagoshima University Graduate School of Medical and Dental Sciences, Kagoshima, Japan; 6 Department of Public Health, Shiga University of Medical Science, Otsu, Japan; 7 Cancer Prevention Center, Chiba Cancer Center Research Institute, Chiba, Japan; 8 Division of Cancer Information and Control, Department of Preventive Medicine, Aichi Cancer Center Research Institute, Nagoya, Japan; 9 Division of Cancer Epidemiology and Prevention, Aichi Cancer Center Research Institute, Nagoya, Japan; 10 Department of Public Health, Nagoya City University Graduate School of Medical Sciences, Nagoya, Japan; 11 Department of Epidemiology for Community Health and Medicine, Kyoto Prefectural University of Medicine, Kyoto, Japan; 12 Department of Psychosomatic Medicine, Kyushu University Graduate School of Medical Sciences, Faculty of Medical Sciences, Fukuoka, Japan; 13 Department of Comprehensive General Internal Medicine, Kyushu University Graduate School of Medical Sciences, Faculty of Medical Sciences, Fukuoka, Japan; 14 Department of Preventive Medicine, Tokushima University Graduate School of Biomedical Sciences, Tokushima, Japan; 15 Laboratory of Public Health, Division of Nutritional Sciences, School of Food and Nutritional Sciences, University of Shizuoka, Shizuoka, Japan; 16 Public Health Informatics Unit, Department of Integrated Health Sciences, Nagoya University Graduate School of Medicine, Nagoya, Japan; 17 Laboratory for Genotyping Development, RIKEN Center for Integrative Medical Sciences, Yokohama, Japan; 18 Department of International and Community Oral Health, Tohoku University Graduate School of Dentistry, Sendai, Japan; 19 Department of Epidemiology, Nagoya University Graduate School of Medicine, Nagoya, Japan

**Keywords:** genome-wide association study, folate metabolism, gene–environment interaction, cardiovascular disease prevention

## Abstract

**Background:**

The present genome-wide association study (GWAS) aimed to reveal the genetic loci associated with folate metabolites, as well as to detect related gene–environment interactions in Japanese.

**Methods:**

We conducted the GWAS of plasma homocysteine (Hcy), folic acid (FA), and vitamin B_12_ (VB_12_) levels in the Japan Multi-Institutional Collaborative Cohort (J-MICC) Study participants who joined from 2005 to 2012, and also estimated gene–environment interactions. In the replication phase, we used data from the Yakumo Study conducted in 2009. In the discovery phase, data of 2,263 participants from four independent study sites of the J-MICC Study were analyzed. In the replication phase, data of 573 participants from the Yakumo Study were analyzed.

**Results:**

For Hcy, *MTHFR* locus on chr 1, *NOX4* on chr 11, *CHMP1A* on chr 16, and *DPEP1* on chr 16 reached genome-wide significance (*P* < 5 × 10^−8^). *MTHFR* also associated with FA, and *FUT2* on chr 19 associated with VB_12_. We investigated gene-environment interactions in both studies and found significant interactions between *MTHFR* C677T and ever drinking, current drinking, and physical activity >33% on Hcy (*β* = 0.039, 0.038 and −0.054, *P* = 0.018, 0.021 and <0.001, respectively) and the interaction of *MTHFR* C677T with ever drinking on FA (*β* = 0.033, *P* = 0.048).

**Conclusion:**

The present GWAS revealed the folate metabolism-associated genetic loci and gene–environment interactions with drinking and physical activity in Japanese, suggesting the possibility of future personalized cardiovascular disease prevention.

## INTRODUCTION

Folic acid (FA) is involved in the transfer of one-carbon units of thymidylate, purines, and methionine.^1^ Intake of FA is essential for DNA synthesis, stability, repair, and normal cell division, especially during rapid growth such as embryonic development and cancer.^1^ The lack of FA in pregnancy is associated with an increased risk of neural tube defects.^2^^,^^3^

Homocysteine (Hcy) is an established blood risk marker of atherosclerosis and subsequent cardiovascular diseases (CVDs). The high concentrations of Hcy are associated with risks of stroke, coronary heart disease, venous thrombosis, peripheral arterial disease, and atherosclerosis.^4^^–^^7^ The increased risk of dementia and Alzheimer’s disease is also associated with elevated Hcy.^8^ The heritability of Hcy is estimated to be 47–70%,^9^^–^^12^ and gene mutations in Hcy metabolic enzymes are well-known to be related to the levels of Hcy.

**Figure 1.**
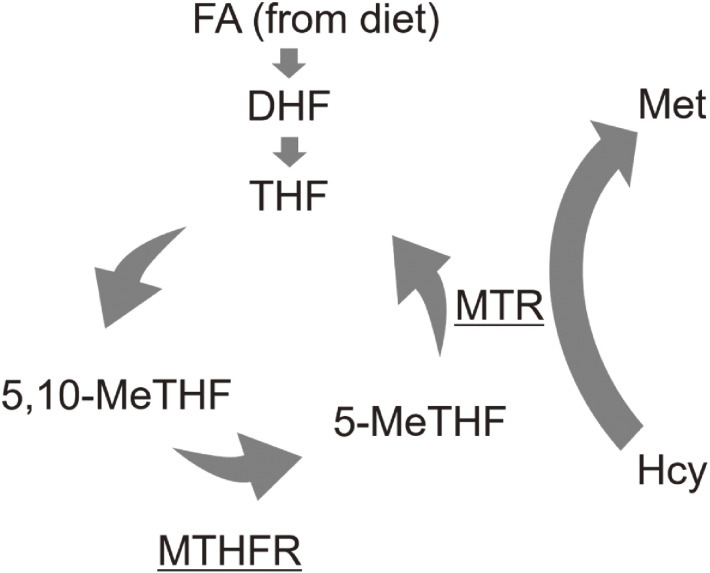
Homocysteine metabolism pathway. Chr, Chromosome; FA, Follic acid; DHF, Dihydrofolate; THF, Tetrahydrofolate; 5,10-MeTHF, 5,10-Methylene THF; 5-MeTHF, 5-Methyl THF; Hcy, Homocysteine; Met, Methionine; MTHFR, Methylenetetrahydrofolate reductase; MTR, 5-Methyltetrahydrofolate-homocysteine methyltransferase.

Vitamin B_12_ (VB_12_) works as a coenzyme for methionine synthase in the formation of Hcy to methionine. The enzyme 5-methyltetrahydrofolate reductase (*MTHFR*) produces 5-methyltetrahydrofolate from 5,10-methyltetrahydrofolate, which is transferred to Hcy by methionine synthase to form methionine and tetrahydrofolate (Figure 1). In Hcy metabolism, FA deficiency and lack of VB_12_ may lead to homocysteinemia, and subsequent vascular inflammation and increased risk of CVDs.^13^ The conditions of the lack of VB_12_ also lead to megaloblastic anemia and neurodegeneration, and to cognitive decline. Moreover, it leads to an increased risk of neural tube defects.^14^

Several studies have reported associations between genetic polymorphisms related to the folate metabolizing pathway and Hcy metabolism. The associations of *MTHFR*, methionine synthase, thymidylate synthase, and serine hydroxylmethyltransferase genes with the risk of several types of cancers, including colorectal cancer, breast cancer, and malignant lymphoma, were found by the candidate single-nucleotide polymorphism (SNP)-based association studies.^15^^–^^18^

In addition, one recent genome wide association study (GWAS) has revealed several functional variants that affect folate metabolisms in humans, including genomic variants in *betaine-homocysteine S-methyltransferase*, *folate hydrolase 1*, and *cystathionine beta-synthase* genes.^19^ Several GWAS on folate metabolisms have also been conducted, but few studies have examined the interaction between these SNPs and lifestyles,^20^ prompting us to investigate these associations to find a way for possible individualized prevention of human disorders related to folate metabolism, such as CVDs, in the future.

This study aimed to investigate the SNPs associated with blood FA, VB_12_, and Hcy levels in Japanese using the GWAS data of the Japan Multi-Institutional Collaborative Cohort (J-MICC) Study and to detect the interactions between genetic polymorphisms and lifestyle variables, such as smoking, alcohol intake, and physical activity (PA), on blood Hcy concentrations, FA, and VB_12_ among Japanese.

## METHODS

### Study subjects

This study is part of the J-MICC study. The J-MICC study is one of the largest genome cohort studies in Japan, conducted at 13 independent universities and research institutions whose main goal is to detect gene–environment interactions mainly for cancer prevention.^21^ The recruitment for participants began in 2005 and ended in March 2014, with a total of 92,610 participants nationwide.^22^ At the baseline survey, the volunteer participants aged 35–69 completed a self-administered questionnaire and provide blood samples after informed consent. We collected blood samples in a 7-mL vacuum tube for serum and a 7-mL EDTA-2Na-containing vacuum tube for plasma and buffy coat. DNA was extracted from buffy coat and provided for genotyping. In the present study, we measured and analyzed plasma Hcy, FA, and VB_12_ levels of 2,263 participants from eight independent study sites of the J-MICC study (Chiba, Aichi Cancer Center, Shizuoka, Daiko, Kyoto, Okazaki, Saga and Kagoshima), who participated in the study from the year of 2005 to 2012.

In the replication phase, we used a single data set consisting of 572 participants from the Yakumo Study.^23^ The Yakumo Study is an epidemiological study conducted annually on health checkup examinees aged ≥39 years who resided in Yakumo-cho, Hokkaido, Japan since the year of 1982.^23^^,^^24^ We asked the 593 health check-up examinees residing in Yakumo-cho, Hokkaido to complete a self-administered questionnaire and provide blood samples after explanation of the aim and the procedure of the study, of whom 572 (96.5%) provided the consent for genetic testing and participated in the study.

Informed consent was obtained from all participants in this study. The protocol of this study was approved by the ethics committees of the Nagoya University Graduate School of Medicine (approval number: 253) and by each participating institution. All research procedures were conducted according to the Ethical Guidelines for Human Genome and Genetic Sequencing Research and the Ethical Guidelines for Medical and Health Research Involving Human Subjects in Japan.

### Sample measurement and phenotype definition

In the J-MICC Study, the plasma sample of participants was stored at −80°C until measurements were performed. The serum Hcy levels were measured with EIA (enzyme immune assay) using JCA-RX20 autoanalyzer in a laboratory (SRL Inc., Hachioji, Japan) in Yakumo Study, and plasma Hcy levels were measured with LC-MS/MS (liquid chromatography-tandem mass spectrometry) in the J-MICC Study; serum FA and VB_12_ levels in Yakumo Study and plasma FA and VB_12_ levels in the J-MICC Study were measured with EIA in the same laboratory. Measurements of Hcy by LC-MS/MS, or the measurements of Hcy, FA and VB_12_ by EIA are shown to have high accuracy with CV (coefficients of variance) within 5%,^25^^–^^28^ suggesting that the results of these measurements are highly reliable. The clinically normal laboratory values are >4.0 ng/mL for plasma FA, 180–914 pg/mL for plasma VB_12_, and 3.7–13.5 nmol/mL for plasma Hcy. In the Yakumo Study, the serum samples of the study participants were measured using the same methods.

### Genotyping, imputation and quality control

DNA samples were automatically extracted from the buffy coat using a BioRobot M48 Workstation (QIAGEN group, Tokyo, Japan). Genotyping for the discovery phase has been performed using the Illumina HumanOmniExpressExome ver1.2 platform (Illumina, San Diego, CA, USA) at the RIKEN Center of the Integrated Medical Sciences (Yokohama, Japan). We estimated identity by descent sharing using the PLINK 1.9 software (https://www.cog-genomics.org/plink2) option ‘- -genome’ to detect closely related pairs and principal component (PC) analysis^29^ with a 1,000 Genomes reference panel (phase 3; http://www.internationalgenome.org/category/phase-3/) using Eigensoft v6.0.1^30^ on an LD pruned SNP set. The LD pruned SNP set was obtained by removing high-LD SNPs with a genotype call rate <0.98, or a minor allele frequency (MAF) <0.01, or Hardy–Weinberg equilibrium exact test *P* value <1 × 10^−6^. The LD pruned SNP set was obtained using the PLINK option ‘- -indep-pair-wise 50 5 0.2’. The identity-by-descent method identified 388 close relationship pairs (pi-hat > 0.1875) and one sample from each of the 388 pairs was deleted. PC analysis with a 1,000 Genomes reference panel (phase 3) found 34 participants whose estimated ancestries were outside the Japanese population, who were identified by visual inspection after plotting the first and second PCs and excluded from the analyses. Finally, filtering for quality control resulted in 14,083 individuals and 575,802 SNPs. Genotype Imputation was Conducted Using SHAPEIT Version 2 (mathgen.stats.ox.ac.uk/genetics_software/shapeit/) and Minimac3 (genome.sph.umich.edu/wiki/Minimac3) software based on the 1,000 Genomes Project cosmopolitan reference panel (phase 3). After the genotype imputation, variants with MAF <0.05 and *r*^2^ < 0.3 were excluded, leaving 6,288,024 variants for final analyses. In the discovery phase, the data for 2,263 subjects of 8 study areas (about 16% of the entire GWAS subjects), whose plasma folate levels were available, were used. In the replication phase, we determined the genotypes of all the discovered SNPs other than the SNP of *NOX4* (*NADPH Oxidase 4*) by the TaqMan real-time polymerase chain reaction (PCR), using the StepOnePlus™ real-time PCR system (Thermofisher, Waltham, MA, USA). As the genotyping for *Nox4* rs2289125 was technically difficult and unsuccessful, we adopted *Nox4* rs10830278 as a surrogate marker, which was in tight linkage with rs2289125 (*D*′ = 0.9792, *r*^2^ = 0.8685). For the *NOX4* rs10830278 polymorphism, we determined the genotypes using PCR with confronting two-pair primers (PCR-CTPP).^31^ The primers used (and the thermal cycler conditions) for *NOX4* rs10830278 were as follows: F1: CTA TTA GGT TGA GCC ATA TAA AAT GGC TGA TT, R1: TCA TGT TGT CAC AAA TGG CAG GA, F2: ATG AGA TTA TAA AAG GGG CCA AGA ACT G and R2: TTG AAT CAT ATA GAT TGG TAG ATC AGA AAC AGT CAA AT (initial denaturation at 95°C for 10 min, followed by 30 cycles of 95°C for 1 min, 58°C for 1 min, and 72°C for 1 min, with a final extension of 72°C for 5 min). The representative gel for the genotyping is shown in eFigure 1. We also confirmed the genotyping results for rs10830278 using PCR-CTPP were completely replicated by TaqMan real-time PCR (primer-probe used: TaqMan SNP Genotyping, SNP ID: C_3223929_10).

### Evaluation of lifestyle information

The lifestyle information was collected using a self-administered questionnaire by well-trained interviewers at the timing of baseline survey. The questionnaire consisted of items on smoking, alcohol consumption, PA, food intake, and medical history.^32^^–^^34^ Smoking habits were categorized as never, former, and current smokers, and pack-years of smoking were counted as well. Alcohol habits were categorized as never, former, and current drinkers, and the amount of alcohol consumption (g/day) was also estimated. Information on PA were collected at baseline survey of J-MICC Study using the questionnaire for the duration per episode in recent years and types of activity. The assigned intensity (in metabolic equivalents) was set based on International Physical Activity Questionnaire as follows^35^^,^^36^: labor work, 4.5; walking, 3.0; mild exercise (not breathtaking), 3.4; breathtaking hard exercise (talkable), 7.0; breathtaking hard exercise (untalkable), 10.0; standing, 2.0; and sitting, 1.5. The total amount of PA was calculated as the sum of the products of duration and intensity for each type of activity in metabolic equivalents * hour/day. PA was dichotomized based on the distribution of the metabolic equivalents * hour/day, which is the well-known unit for metabolic equivalent, where subjects were coded as 1 if their PAs were more than or equal to 33 percentile, and coded as 0 otherwise.

### Statistical analysis

We examined the associations of the SNPs with the quantitative traits of plasma FA, VB_12_, and Hcy using the EPACTS software (http://genome.sph.umich.edu/wiki/EPACTS). The associations of SNPs with the natural logarithms of plasma FA, VB_12_, and Hcy as continuous variables were tested using linear regression. For covariates to be adjusted, gender, age, and the first five principal components were included. All 6,288,024 variants with the MAF greater than (or equal to) 0.05 were considered. Manhattan and Q-Q Plots were generated using the ‘qqman’ function in R (https://cran.r-project.org/web/packages/qqman/index.html). Lead SNPs (or variants) were defined as those SNPs (or the variants) that reached the minimum *P*-values in each genetic locus, defined as the positions on the chromosome identified by the cytogenetic banding of the chromosome.^37^ The *β* coefficient for the gene–environment interaction was estimated based on linear regression with the multiplicative product term for the interaction of the number of minor alleles and lifestyles as binary variables. The GxEs of GWAS identified SNPs with lifestyles of smoking, alcohol consumption and physical activity on blood levels of folate metabolites (Hcy, FA and VB12) were examined.

For the analyses of GWAS, the genome-wide significance levels were set at *P* < 5 × 10^−8^, and suggestive levels were set at *P* < 1 × 10^−6^; for the rest of the analyses, statistical significance levels were set at *P* < 0.05, where adjustments for multiple comparisons were not applied due to the exploratory nature of the analyses.^38^

## RESULTS

### Study characteristics and the genome-wide association study of folate metabolites

The characteristics of the study participants were shown in Table 1. In the J-MICC Study, median plasma Hcy concentration were higher in men than in women (8.8; interquartile range [IQR], 7.2–10.8 nmol/mL vs 6.8; IQR, 5.8–8.2 nmol/mL, respectively; *P* < 0.001), but median plasma FA and VB_12_ were higher in women than in men (855; IQR, 730–1,010 pg/mL vs 800; IQR, 700–945 pg/mL, respectively; *P* < 0.001).

**Table 1.** Characteristics of participants in the Japan Multi-Institutional Collaborative Cohort (J-MICC)

	J-MICC							Yakumo						
	
	Total (*n* = 2,263)		Men (*n* = 1,036)		Women (*n* = 1,227)		*P* value	Total (*n* = 572)		Men (*n* = 212)		Women (*n* = 360)		*P* value
Age, years, mean SD	55.7	8.9	56.6	8.7	54.9	9.0	<0.001	64.4	10.2	66.6	10.4	63.1	9.9	<0.001
FA, ng/mL, median (IQR)	8.5	(7.4–10.1)	8.1	(7.1–9.4)	8.9	(7.7–10.5)	<0.001	5.8	(4.6–7.8)	5.3	(4.1–7.0)	6.1	(4.9–8.1)	0.018
Hcy, nmol/mL, median (IQR)	7.6	(6.2–9.4)	8.8	(7.2–10.8)	6.8	(5.8–8.2)	<0.001	7.5	(6.5–9.3)	8.7	(7.3–10.6)	7.0	(6.2–8.2)	<0.001
VB_12_, pg/mL, median (IQR)	835	(715–975)	800	(700–945)	855	(730–1,010)	0.931	1,070	(829–1,280)	1,035	(804–1,245)	1,110	(845–1,305)	0.174
Smoking, *n* (%)														
Never	1,346	(59.5)	268	(25.9)	1,078	(87.9)	<0.001	369	(64.5)	69	(32.5)	300	(83.3)	<0.001
Former	507	(22.4)	449	(43.3)	58	(4.7)		119	(20.8)	91	(42.9)	28	(7.8)	
Current	408	(18.0)	318	(30.7)	90	(7.3)		84	(14.7)	52	(24.5)	32	(8.9)	
Former + current	915	(40.4)	767	(74.0)	148	(12.1)		203	(35.5)	143	(67.4)	60	(16.7)	
Pack-years, mean SD	16.8	28.9	33.5	34.7	2.8	9.4		32.7	20.0	39.0	19.9	22.5	15.6	
Drinking, *n* (%)														
Never	932	(41.2)	188	(18.2)	744	(60.6)	<0.001	302	(52.8)	51	(24.1)	251	(69.7)	<0.001
Former	39	(1.7)	24	(2.3)	15	(1.2)		20	(3.5)	13	(6.1)	7	(1.9)	
Current	1,290	(57.0)	823	(79.4)	467	(38.1)		250	(43.7)	148	(69.8)	102	(28.3)	
Former + current	1,329	(58.7)	847	(81.8)	482	(39.3)		270	(47.2)	161	(75.9)	109	(30.2)	
Alcohol consumption, g/day, mean SD	15.5	39.5	15.7	40.9	15.3	38.2		24.7	28.5	33.5	31.4	11.8	16.7	

FA, folic acid; Hcy, homocysteine; VB12, vitamin B12.

**Figure 2.**
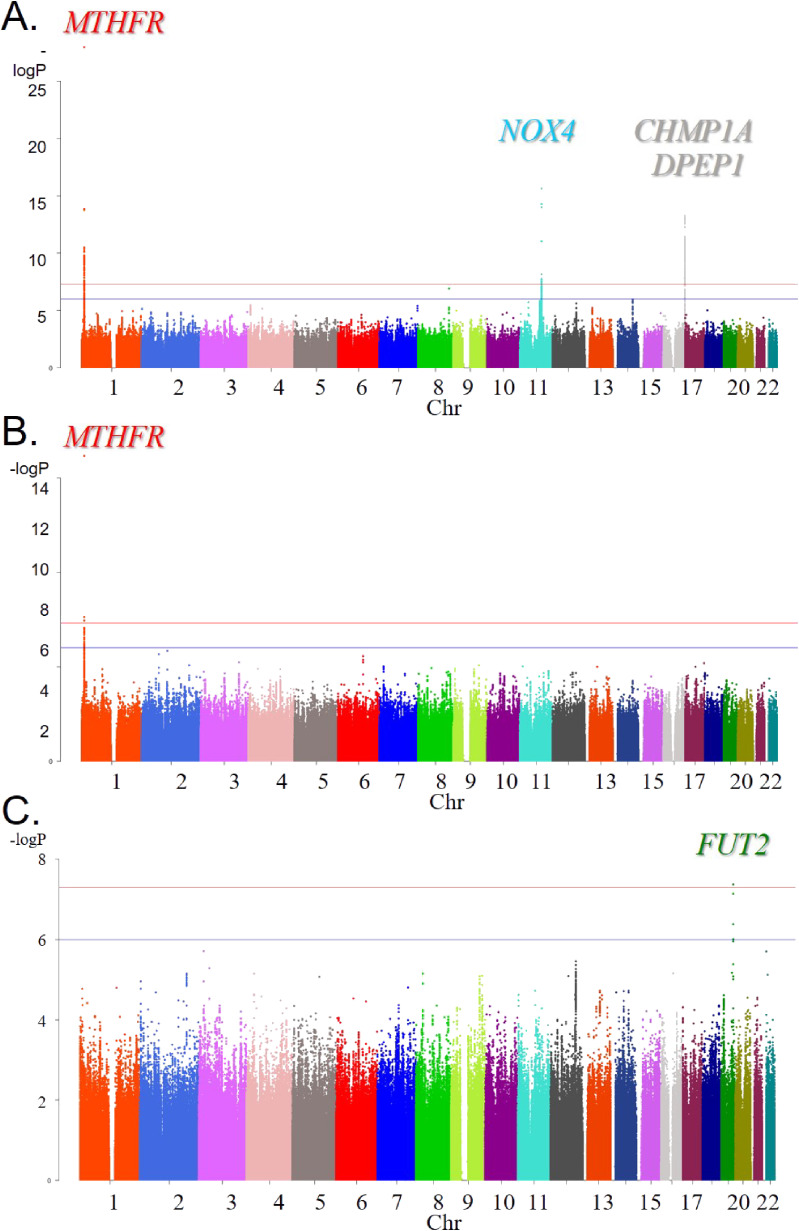
Manhattan Plots for the genome-wide association study (GWAS) of plasma folate metabolites of homocysteine (Hcy)/folic acid (FA)/vitamin B_12_ (VB_12_) in the Japan Multi-Institutional Collaborative Cohort (J-MICC) ([**A**] Hcy: *n* = 2,192; [**B**] FA: *n* = 2,263; [**C**] VB_12_: *n* = 2,260).

**Figure 3.**
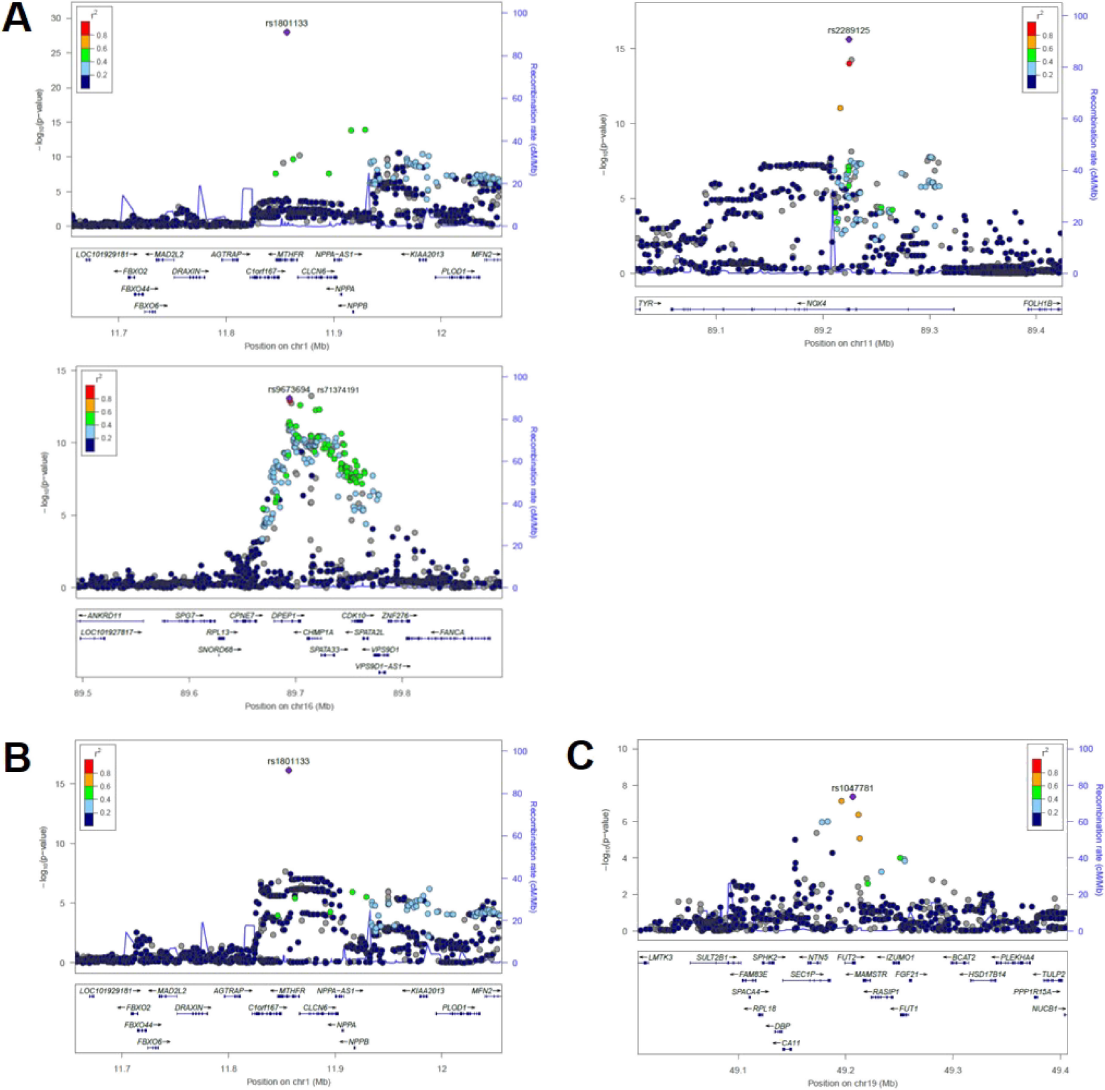
Regional Plots for the Genome Wide Association Study (GWAS) of plasma folate metabolites of homocysteine (Hcy)/folic acid (FA)/vitamin B_12_ (VB_12_) in the Japan Multi-Institutional Collaborative Cohort (J-MICC) ([**A**] Hcy: *MTHFR* locus on 1p36, *NOX4* locus on 11q14 and *DPEP1-CHMP1A* locus on 16q24; [**B**] FA: *MTHFR* locus on 1p36; [**C**] VB_12_: *FUT2* locus on 19q13).

At the first stage of GWAS, we found that multiple SNPs were associated with plasma Hcy, FA, and VB_12_ levels at genome-wide significant level of *P* < 5 × 10^−8^ in six genomic regions (Table 2). The Manhattan plots, regional plots for the gene locus found to be genome-wide significant and Q-Q plots for the GWAS of Hcy, FA, and VB_12_ levels are shown in Figure 2, Figure 3, and eFigure 2, respectively. Plasma Hcy levels were significantly associated with three loci; *MTHFR* locus on chr 1 (lead SNP: rs1801133, also known as *MTHFR* C677T with *β* = 0.09178 and *P* = 1.10 × 10^−28^), *NOX4* locus on chr 11 (rs2289125 with *β* = 0.06557 and *P* = 2.28 × 10^−16^), *charged multivesicular body protein 1A* (*CHMP1A*) on chr 16 (rs71374191 with *β* = 0.06382 and *P* = 5.84 × 10^−14^), and *dipeptidase 1* (*DPEP1*) on chr 16 (rs9673694 with *β* = 0.06481 and *P* = 8.38 × 10^−14^, and rs1126464 with *β* = −0.06293 and *P* = 2.67 × 10^−13^), both of which are considered as located on the same *DPEP1/CHMP1A* genomic locus.^39^ Plasma FA levels were associated with *MTHFR* locus on chr 1 (lead SNP: rs1801133 with *β* = −0.05960 and *P* = 7.05 × 10^−17^), and plasma VB_12_ levels were associated with fucosyltransferase 2- (*FUT2*) locus on chr 19 (rs1047781 with *β* = 0.04298 and *P* = 4.30 × 10^−8^) at genome-wide significant level of *P* < 5 × 10^−8^. In the independent dataset of Yakumo Study, most of the GWAS significant loci were in the same direction of effect (*MTHFR* rs1801133 with *β* = 0.02519 and *P* = 0.106, *NOX4* rs2289125 with *β* = 0.00391 and *P* = 0.821, *CHMP1A* rs71374191 with *β* = 0.02780 and *P* = 0.013, and *DPEP1* rs9673694 with *β* = 0.02264 and *P* = 0.166), except for the *DPEP1* rs1126464 (*β* = −0.02260 and *P* = 0.339) and *FUT2* rs1047781 (*β* = −0.11459 and *P* < 0.001) (Table 2). In addition, we also conducted the GWAS of folate metabolites adjusted for FA intake, which did not substantially affect the results (data not shown). For the suggestively significant loci (*P* < 1 × 10^−6^), the *PVT1* locus on chromosome 8 associated with plasma Hcy had never been reported based on the information of GWAS catalogue (www.ebi.ac.uk/gwas/), suggesting that this SNPs was newly found (eTable 2).

**Table 2.** The significant SNPs associated with plasma homocysteine (Hcy)/folic acid (FA)/vitamin B_12_ (VB_12_) level from Genome Wide Association Study (GWAS) of participants in the Japan Multi-Institutional Collaborative Cohort (J-MICC)

Hcy												

									J-MICC		Yakumo	
	
rsID	Genotyped/ imputed	Cytoband	Chr	Position	Gene	Function	Effect allele	Ref. allele	Beta	*P* value	Beta	*P* value
rs1801133	genotyped	1p36.22	1	11856378	*MTHFR*	Nonsynonymous	*A*	*G*	0.09178	1.10 × 10^−28^	0.02519	0.106
rs2289125	imputed	11q14.3	11	89224453	*NOX4*	Utr5	*C*	*A*	0.06557	2.28 × 10^−16^	0.00391	0.821
rs71374191	imputed	16q24.3	16	89714866	*CHMP1A*	Intron	*C*	*T*	0.06382	5.84 × 10^−14^	0.02870	0.013
rs9673694	imputed	16q24.3	16	89694169	*DPEP1*	Intron	*C*	*A*	0.06481	8.38 × 10^−14^	0.02264	0.166
rs1126464	genotyped	16q24.3	16	89704365	*DPEP1*	Nonsynonymous	*C*	*G*	−0.06293	2.67 × 10^−13^	0.02671	0.103


FA												

									J-MICC		Yakumo	
	
rsID	Genotyped/ imputed	Cytoband	Chr	Position	Gene	Function	Effect allele	Ref. allele	Beta	*P* value	Beta	*P* value

rs1801133	genotyped	1p36.22	1	11856378	*MTHFR*	Nonsynonymous	*A*	*G*	−0.05960	7.05 × 10^−17^	−0.02260	0.339


VB_12_												

									J-MICC		Yakumo	
	
rsID	Genotyped/ imputed	Cytoband	Chr	Position	Gene	Function	Effect allele	Ref. allele	Beta	*P* value	Beta	*P* value

rs1047781	imputed	19q13.33	19	49206631	*FUT2*	Nonsynonymous	*T*	*A*	0.04298	4.30 × 10^−8^	−0.11459	<0.001

Hcy, homocysteine; *MTHFR*, 5-methyltetrahydrofolate reductase; *NOX4*, NADPH oxidase 4; *CHMP1A*, charged multivesicular body protein 1A; *DPEP1*, dipeptidase 1; FA, folic acid; VB_12_, vitamin B_12_; *FUT2*, fucosyltransferase 2.

### Gene–environment interaction analyses

We also examined the gene–environment interactions of *MTHFR C677T* (rs1801133) with lifestyles (Table 3). Although the previously reported interaction of *MTHFR C677T* and smoking habits on plasma Hcy was only marginal (*β* = 0.029, *P* = 0.089), a significant interaction of *MTHFR C677T* and drinking habits on plasma FA, and the interaction of *MTHFR C677T* and PA on plasma Hcy were observed (*β* = −0.040, *P* = 0.020; *β* = 0.035, *P* = 0.040) (eFigure 3).

**Table 3.** The interactions of *MTHFR* C677T rs1801133 and lifestyles on plasma folate metabolites of homocysteine (Hcy)/folic acid (FA)/vitamin B_12_ (VB_12_) with lifestyle in the Japan Multi-Institutional Collaborative Cohort (J-MICC) and Yakumo study

		*MTHFR*					
		rs1801133					

		J-MICC		Yakumo Study		Meta	
		
		Beta^a^	*P*-value^a^	Beta^a^	*P*-value^a^	Beta^a^	*P*-value^a^
Smoking							
Hcy							
	ever	0.029	0.089	−0.012	0.706	0.020	0.184
	current	0.030	0.185	−0.082	0.048	0.004	0.822
FA							
	ever	0.016	0.270	0.035	0.486	0.018	0.209
	current	0.015	0.445	0.009	0.887	0.014	0.440
VB12							
	ever	−0.028	0.124	0.020	0.699	−0.022	0.186
	current	−0.037	0.107	0.032	0.636	−0.030	0.171
Drinking							
Hcy							
	ever	0.034	0.080	−0.004	0.903	0.024	0.156
	current	0.033	0.151	−0.006	0.088	0.023	0.175
FA							
	ever	0.035	0.040	0.058	0.220	0.037	0.019
	current	0.033	0.129	0.074	0.054	0.037	0.020
VB12							
	ever	−0.032	0.120	−0.023	0.636	−0.031	0.106
	current	−0.034	0.086	−0.020	0.102	−0.032	0.095

PA							
Hcy							
	PA >33 percentile	−0.040	0.020	−0.054	0.100	−0.043	0.005

*MTHFR*, 5-methyltetrahydrofolate reductase; J-MICC, Japan Multi-Institutional Collaborative Cohort; Hcy, homocysteine; FA, folic acid; VB_12_, vitamin B_12_; PA, physical activity.

^a^Adjusted for age and sex.

To validate these findings, we examined the corresponding interactions in an independent dataset from the Yakumo Study (Table 3). While only statistically marginal, we recapitulated the trend of the interactions of *MTHFR* 677 *T/T* with current drinking on FA and with PA on Hcy (*β* = 0.074, *P* = 0.054, and *β* = −0.054, *P* = 0.100, respectively).

We also examined these observed interactions in a combined data set of J-MICC and Yakumo Study. A significant interaction between *MTHFR* C677T and drinking habits on plasma FA (*β* = 0.037, *P* = 0.019 for the interaction of *MTHFR* C677T and ever drinking, *β* = 0.037, *P* = 0.020 for the interaction with current drinking), and the interaction between *MTHFR* C677T and PA on plasma Hcy were observed (*β* = −0.043, *P* = 0.005). (Table 3). In addition, the exhaustive examination of gene–environment interactions between the lead SNPs (and the surrogate, *Nox4* rs10830278) found in the present GWAS and the lifestyles of smoking habits, drinking habits, and PA revealed several statistically significant associations, although most of the interactions based on smoking, drinking, or PA as continuous variables failed to reach any statistical significance, which may worth verifying in future studies (eTable 1).

### Associations of folate metabolites with the reported loci

We also examined the associations of each of folate metabolites (Hcy, FA, and VB12) with previously reported loci based on GWAS catalogue (trait ID: EFO_0004578, EFO_0005111 and EFO_0004620). As a result, statistically significant associations of rs1801133 (in the *MTHFR* gene on chromosome 1), rs12085006, and rs1999594 (both on chromosome 1) with blood Hcy levels and that of rs1801133 with blood FA levels were observed (eTable 3).

## DISCUSSION

Polymorphisms of *MTHFR*, *DPEP1*, *NOX4*, and *FUT2* have been previously reported to be associated with FA, VB_12_, and Hcy levels.^20^^,^^40^^,^^41^ However, no GWAS of the folate metabolic pathways has been reported in Japanese. The present GWAS suggested that polymorphisms of *MTHFR*, *DPEP1*, *CHMP1A*, *FUT2*, and *NOX4* may play an important role in the regulation of plasma Hcy levels in Japanese.

Hcy remethylation requires a cosubstrate 5-methyltetrahydrofolate to form methionine. *MTHFR* catalyzes the reduction of 5,10-methylenetetrahydrofolate to 5-methyltetrahydrofolate.^42^^–^^44^ The homozygotes of the thermolabile *MTHFR* 677*T* allele are shown to have reduced *MTHFR* enzyme activity,^45^ which leads to higher blood Hcy levels and subsequent elevated risks of various vascular/atherosclerotic diseases.^6^

*NOX4* has been previously associated with Hcy in three studies.^46^^–^^48^ *NOX4* is expressed in endothelial cells, cardiomyocytes, and vascular smooth muscle cells,^49^ and elevated expressions were reported in patients with hypertension, atherosclerosis, heart failure, and stroke.^50^ Upregulation of *NOX4* may also contributes to Hcy-mediated apoptosis of endothelial cells.^51^

*DPEP1* is a kidney membrane enzyme that is highly expressed in the proximal convoluted tubules.^52^ Although their roles in Hcy metabolism remain unclear, they may be involved in renal handling and metabolism of Hcy and cysteine, a precursor of Hcy. *DPEP1* was previously associated with Hcy in one analysis.^48^

We also found a reported association between *CHMP1* polymorphism and plasma Hcy in humans.^47^ *CHMP1A* is involved in protein transport, and is essential for the proliferation and maintenance of neural progenitor cells.^53^

*FUT2* (rs602662, rs492602, and the *FUT2* haplotype) was associated with VB_12_ levels, which confirmed previously reported findings.^19^^,^^54^^,^^55^ Another non-synonymous SNP in the *FUT2* gene rs10447781 was strongly associated with VB_12_ levels. *FUT2* variants reportedly reduce H-type antigen production and function and decrease the risk of VB_12_ malabsorption due to *Helicobacter pylori* infection and associated gastritis.^56^ In addition, *FUT2* variants increase the secretion of fucosylated glycoprotein of gastric intrinsic factors required for VB_12_ absorption.^57^

The functionality of *MTHFR* rs1801133 was already well demonstrated in previous reports^45^; the rs2289125 SNP of *NOX4* is located in the open chromatin in most tissues and thus may associate with transcription regulation.^46^ The rs1126464 SNP of *DPEP1* and the rs1047781 of *FUT2* is located in the non-synonymous region and may exert effect through alteration of *DPEP1* protein structure.

Some of the SNPs found in the present GWAS in association with blood Hcy, FA, and VB12 compared with previously found SNPs in the GWAS were partially the same (rs1801133 of *MTHFR* and rs2289125 of *NOX4*) but other SNPs were different SNPs in the same genes/genomic loci. For example, the and rs71374191 of the *DPEP1/CHMP1A* locus found in association with blood Hcy levels were different from those reported in European GWAS (rs7130284 of *NOX4* and rs154657 of *DPEP1*).^47^ Although the present study found the rs1047781 of *FUT2* as the top significant SNP for VB_12_, the two SNPs of rs602662 and rs492602 of *FUT2* are non-polymorphic in Japanese. The different SNPs found to be GWAS significant in association with blood folate metabolites may suggest the results of different evolutionary pressure between ethnicities and may lead to population-specific prevention strategies against diseases related to folate metabolism disorders, such as atherosclerosis and CVDs.^58^^,^^59^

In previous studies, a positive interaction between *MTHFR* C677T and smoking habits on plasma Hcy was reported.^18^^,^^60^^–^^63^ The present study revealed the marginal interaction in the same direction, although it did not reach statistical significance. According to the Organisation for Economic Co-operation and Development (OECD) health statistics (https://stats.oecd.org/), the rate of male smokers is higher in Japan (around 30%) compared with those in other OECD member countries, such as United States or United Kingdom (around 10–20%). The interaction with smoking habits for *MTHFR* and vascular diseases risk may be explained by a similar mechanism via elevated Hcy.^63^ Considering the different prevalence of smokers between countries, further investigation with sufficiently large sample sizes in each country’s population is needed to clarify the interaction of *MTHFR* C677T with smoking habits on blood Hcy levels.

This study observed a positive interaction between *MTHFR* C677T and drinking habits on blood FA (ie, as the *T* allele of *MTHFR* C677T and drinking amount increase, they have multiplicative interactive effects on the increment of FA), and a negative interaction between *MTHFR* C677T and PA on blood Hcy (ie, as the *T* allele of *MTHFR* C677T and calories spent by PA increase, they have multiplicative interactive effects on the reduction of Hcy) in Japanese. *MTHFR* 677*T/T* is a genotype similar to *ALDH2 Lys/Lys*, and individuals with *MTHFR* 677*T/T* may have avoidant and deterrent responses to alcohol consumption due to excessive savings of Hcy in those with *MTHFR* 677*T/T*, which may explain the possible gene–environment interaction between drinking habits and *MTHFR* 677*T* allele on blood FA levels.^64^ This might act against alcohol dependence and lead to attenuated FA reduction in those with *MTHFR* 677*T/T* genotype in drinkers, which is consistent with the results of previous studies.^20^^,^^65^ In the present study, however, no significant association between *MTHFR* 677*T/T* genotype and drinking behavior was observed. Considering that the recent meta-analysis revealed no association of *MTHFR* 677*T/T* and alcohol dependence, the effect of *MTHFR* genotype on drinking behavior might be relatively limited.^66^ In addition, the reduced blood FA levels in subjects with *MTHFR* 677 *T/T* genotype in the present study are in accordance with the previous report,^67^ underscoring the requirements for more folate intake in those with *MTHFR* 677 *T/T* genotype than in those with other genotypes. The influence of *MTHFR* polymorphism on the association between PA and blood Hcy levels has been an issue of interest to researchers in recent decades.^65^^,^^68^^,^^69^ This study might add some beneficial evidence for possible personalized prevention of atherosclerosis/CVDs based on genetic information in the near future. The detailed mechanisms of the reduction in plasma Hcy levels with exercise are not yet well described, which may be explained by mechanisms of protein turnover and the betaine pathway.^70^^,^^71^ The existence of different distributions of lifestyle factors, as well as different genotype distributions of SNPs, may lead to discovery of different GxEs and disease prevention measures specific to each population. Further investigations with sufficiently larger sample sizes in each population should be expected to clarify these associations.

The strength of the present study would be that this GWAS was conducted using only population-based cohort data from Japanese population with relatively large sample size, so it is possible that gene–environment interactions might be easier to detect without adjustment in Japanese population, where lifestyle components are relatively homogeneous.

Our study has several limitations. First, all lifestyle factors are self-reported and thus subject to bias, which is generally bias toward null. Second, we investigated the gene–environment interaction based only on the SNPs detected from GWAS results of folates (Hcy, FA, and VB_12_) as outcomes. Although there is an alternative method to examine gene–environment interactions comprehensively based on GWAS data using the ProbABEL, the gene–environment interaction detection software in GWAS, we adopted a candidate SNP approach using GWAS detected SNPs because GWAS data using current folate measurement data (*n* = 2,263) are somewhat underpowered to detect robust gene–environment interactions. In addition, the cut-off level for the dichotomization of PA was determined to be 33% based on our sensitivity analysis for different cut-off levels of PA, which was mainly due to the exploratory context of the present study. Further investigations with sufficiently large sample sizes for this potentially intriguing interaction of *MTHFR* 677*T/T* with PA on blood Hcy levels should be warranted.

### Conclusion

The present GWAS revealed the significance of folate metabolism-associated SNPs in *MTHFR*, *DPEP1*, *CHMP1*, and *NOX4* genes also in Japanese. In addition, the interactions of these SNPs with lifestyles of smoking, drinking and PA on blood folate metabolite concentrations may pave the way for the possible personalized prevention of atherosclerotic diseases in Japanese, as well as in world populations. Further investigations are needed to confirm the results of the present study.

## SUPPLEMENTARY MATERIAL

eFigure 1. Representative gel for the genotyping of NADPH oxidase 4 (*Nox4*) rs10830278. Genotyping for *Nox4* rs10830278: Lane M, 100-bp marker; lane 1, *T/T* genotype (228-bp and 297-bp bands); lane 2, *A/T* genotype (138-bp, 228-bp and 297-bp bands); lane 3, *A/A* genotype (138-bp and 297-bp bands).

eFigure 2. Q-Q Plots for the Genome Wide Association Study (GWAS) of plasma folate metabolites of homocysteine (Hcy)*/*folic acid (FA)*/*vitamin B_12_ (VB_12_) in the Japan Multi-Institutional Collaborative Cohort (J-MICC) ([**A**] Hcy: n = 2,192; [**B**] FA: n = 2,263; [**C**] VB_12_: n = 2,260).

eFigure 3. Gene–environment interaction of 5-methyltetrahydrofolate reductase (*MTHFR*) *C677T* with lifestyle factors on plasma folate metabolites ([**A**] *MTHFR C677T* and ever drinking on folic acid [FA]; [**B**] *MTHFR C677T* and physical activity (PA) >33 percentile on Hcy).

eTable 1. The interactions of SNPs with smoking and alcohol intake on plasma folate metabolites of homocysteine (Hcy)/folic acid (FA)/vitamin B_12_ (VB_12_) in the combined data of the Japan Multi-Institutional Collaborative Cohort (J-MICC) and Yakumo study

eTable 2. Suggestively significant SNPs associated with plasma homocysteine (Hcy)/folic acid (FA)/vitamin B12 (VB12) level from Genome Wide Association Study (GWAS) of participants in the Japan Multi-Institutional Collaborative Cohort (J-MICC)

eTable 3. Associations of folate metabolites with the reported loci
