# Supplementary material for: GWAS of Folate Metabolism With Gene–environment Interaction Analysis Revealed the Possible Role of Lifestyles in the Control of Blood Folate Metabolites in Japanese: The J-MICC Study
Source: J Epidemiol. 2024 May 5;34(5):228–37. doi: 10.2188/jea.JE20220341 (PMC10999522; doi:10.2188/jea.JE20220341)
Supplement: Supplementary file 1 [file je-34-228-s001.pdf]

eTable 1. The interactions of SNPs with smoking and alcohol intake on plasma folate metabolites of homocysteine (Hcy)/folic acid (FA)/vitamin B12 (VB12) in the combined data of the Japan Multi-Institutional Collaborative Cohort (J-MICC) and Yakumo study.

eTable 2. Suggestively significant SNPs associated with plasma homocysteine (Hcy)/folic acid (FA)/vitamin B12 (VB12) level from Genome Wide Association Study (GWAS) of participants in the Japan Multi-Institutional Collaborative Cohort (J-MICC).

eTable 3. Associations of folate metabolites with the reported loci.

eTable 1. The interactions of SNPs with smoking and alcohol intake on plasma folate metabolites of homocysteine (Hcy)/folic acid (FA)/vitamin B_{12} (VB_{12}) in the combined data of the Japan Multi-Institutional Collaborative Cohort (J-MICC) and Yakumo study

	NOX4		*CHMP1A*		*DPEP1* (non-synonym)		*DPEP1* (intron)		*FUT2*	
	rs10830278		rs71374191		rs1126464		rs9673694		rs1047781	
	Beta[a]	*P*-value[a]	Beta[a]	*P*-value[a]	Beta[a]	*P*-value[a]	Beta[a]	*P*-value	Beta[a]	*P*-value[a]
Smoking										
Hcy										
ever	-0.001	0.958	-0.008	0.569	-0.006	0.694	-0.015	0.348	-0.005	0.730
current	0.007	0.723	-0.040	0.031	-0.039	0.060	-0.047	0.027	-0.012	0.566
FA										
ever	0.021	0.128	0.020	0.166	0.017	0.245	0.010	0.520	-0.016	0.274
current	0.033	0.075	0.012	0.508	-0.009	0.657	-0.011	0.562	-0.028	0.148
VB_{12}										
ever	-0.029	0.072	0.059	0.109	0.042	0.017	0.039	0.031	-0.008	0.650
current	-0.034	0.119	0.034	0.109	0.040	0.080	0.039	0.096	-0.014	0.545
Drinking										
Hcy										
ever	0.002	0.887	-0.030	0.206	-0.039	0.027	-0.034	0.059	-0.039	0.023
current	0.001	0.963	-0.031	0.048	-0.041	0.022	-0.034	0.062	-0.043	0.013
FA										
ever	0.017	0.255	0.002	0.880	-0.006	0.723	-0.008	0.636	0.033	0.045
current	0.013	0.407	0.002	0.918	0.022	0.258	-0.009	0.623	-0.030	0.121

VB_{12}											
	ever	-0.012	0.491	0.026	0.158	0.022	0.258	0.016	0.450	-0.030	0.121
	current	-0.012	0.484	0.024	0.193	0.021	0.286	0.013	0.529	-0.025	0.194
PA											
Hcy >33 percentile		0.012	0.405	0.037	0.011	0.039	0.016	0.050	0.003	0.005	0.757

NOX4, *NADPH oxidase 4*; *CHMP1A*, *charged multivesicular body protein 1A*; *DPEP1*, *dipeptidase 1*; *FUT2*, *fucosyltransferase 2*; Hcy, homocysteine; FA, folic acid; VB_{12}, vitamin B_{12}; PA, physical activity.

[a]Adjusted for age and sex.

eTable 2. Suggestively significant SNPs associated with plasma homocysteine (Hcy)/folic acid (FA)/vitamin B12 (VB12) level from Genome Wide Association Study (GWAS) of participants in the Japan Multi-Institutional Collaborative Cohort (J-MICC)

Hcy

rsID	Genotyped/ imputed	Chr	MARKER_ID	Position	N	Beta	t	*P*-value
rs1801133	genotyped	1	1:11856378_G/A_Nonsynonymous:MTHFR	11856378	2,192	0.091782	11.273	1.10E-28
rs2289125	imputed	11	11:89224453_A/C_Utr5:NOX4	89224453	2,192	0.065569	8.2713	2.28E-16
rs71374191	imputed	16	16:89714866_T/C_Intron:CHMP1A	89714866	2,192	0.063819	7.5615	5.84E-14
rs9673694	imputed	16	16:89694169_A/C_Intron:DPEP1	89694169	2,192	0.064807	7.5132	8.38E-14
rs1126464	genotyped	16	16:89704365_G/C_Nonsynonymous:DPEP1	89704365	2,192	-0.06293	-7.356	2.67E-13
-	imputed	1	1:11868284_TTGTG/T_Deletion:CLCN6	11868284	2,192	0.053957	6.5664	6.43E-11
rs6684539	imputed	1	1:11968273_A/G_Exon:RNU5E-1	11968273	2,192	-0.0524	-6.3164	3.24E-10
rs4993565	imputed	1	1:11969993_A/G_Upstream:RNU5E-4P	11969993	2,192	-0.05152	-6.1492	9.24E-10
rs258322	genotyped	16	16:89755903_A/G_Intron:CDK10	89755903	2,192	-0.0491	-6.0361	1.85E-09
rs3209284	imputed	1	1:11979909_T/C_Utr3:KIAA2013	11979909	2,192	-0.05	-5.9649	2.85E-09
rs3751695	imputed	16	16:89764549_G/A_Synonymous:SPATA2L	89764549	2,192	-0.05219	-5.761	9.55E-09
rs3737961	imputed	1	1:12033113_A/G_Intron:PLOD1	12033113	2,192	-0.04534	-5.5079	4.06E-08
rs6677365	imputed	1	1:12047688_G/A_Intron:MFN2	12047688	2,192	-0.0445	-5.3955	7.57E-08
rs12680310	imputed	8	8:128932807_C/T_Intron:PVT1	1.29E+08	2,192	0.048161	5.3053	1.24E-07

-	imputed	1	1:12047196_G/GTC_Insertion:MFN2	12047196	2,192	-0.04366	-5.2978	1.29E-07

FA

rsID	Genotyped/ imputed	Chr	MARKER_ID	Position	N	Beta	t	*P*-value
rs1801133	genotyped	1	1:11856378_G/A_Nonsynonymous:MTHFR	11856378	2,263	-0.0596	-8.4123	7.05E-17
rs138473600	imputed	1	1:11880755_C/T_Utr3:CLCN6	11880755	2,263	0.063944	5.3644	8.96E-08

VB12

rsID	Genotyped/ imputed	Chr	MARKER_ID	Position	N	Beta	t	*P*-value
rs1047781	imputed	19	19:49206631_A/T_Nonsynonymous:FUT2	49206631	2,260	0.042984	5.4971	4.30E-08
rs3826837	imputed	19	19:49183226_G/A_Intron:SEC1P	49183226	2,260	0.039458	4.9099	9.77E-07

eTable 3. Associations of folate metabolites with the reported loci

Hcy

rsID	Genotyped/ imputed	Chr	MARKER_ID	Position	N	MAF	Beta	SE	t	*P*-value
rs1801133	imputed	1	1:11856378_G/A_1:11856378	11856378	2,192	0.37819	0.091782	0.008142	11.273	1.10E-28
rs12085006	imputed	1	1:11958723_A/G_1:11958723	11958723	2,192	0.40146	-0.04894	0.008248	-5.9341	3.43E-09
rs1999594	genotyped	1	1:11959216_G/A_1:11959216	11959216	2,192	0.40214	-0.04843	0.008249	-5.8707	5.01E-09
rs730123	genotyped	1	1:12070292_G/A_1:12070292	12070292	2,192	0.46761	-0.03418	0.007975	-4.2865	1.89E-05
rs2275565	genotyped	1	1:237048676_G/T_1:237048676	237048676	2,192	0.24589	0.008822	0.009437	0.93487	0.34996

FA

rsID	Genotyped/ imputed	Chr	MARKER_ID	Position	N	MAF	Beta	SE	t	*P*-value
rs1801133	genotyped	1	1:11856378_G/A_1:11856378	11856378	2,263	0.37892	-0.0596	0.007085	-8.4123	7.05E-17
rs13306561	genotyped	1	1:11865804_A/G_1:11865804	11865804	2,263	0.096111	0.063769	0.01191	5.3543	9.46E-08
rs2639453	genotyped	1	1:11982544_T/C_1:11982544	11982544	2,263	0.33451	0.035127	0.007416	4.737	2.30E-06
rs1999594	genotyped	1	1:11959216_G/A_1:11959216	11959216	2,263	0.40013	0.032486	0.007122	4.5611	5.36E-06
rs2840534	genotyped	1	1:2303566_A/G_1:2303566	2303566	2,263	0.096774	-0.0034	0.011708	-0.2904	0.77154
rs10916232	genotyped	1	1:228058967_G/A_1:228058967	228058967	2,263	0.1973	0.001012	0.008731	0.11591	0.90773

VB12

rsID	Genotyped/ imputed	Chr	MARKER_ID	Position	N	MAF	Beta	SE	t	*P*-value
rs7544372	imputed	1	1:240987754_C/T_1:240987754	240987754	2,260	0.35664	0.008149	0.007796	1.0452	0.29602

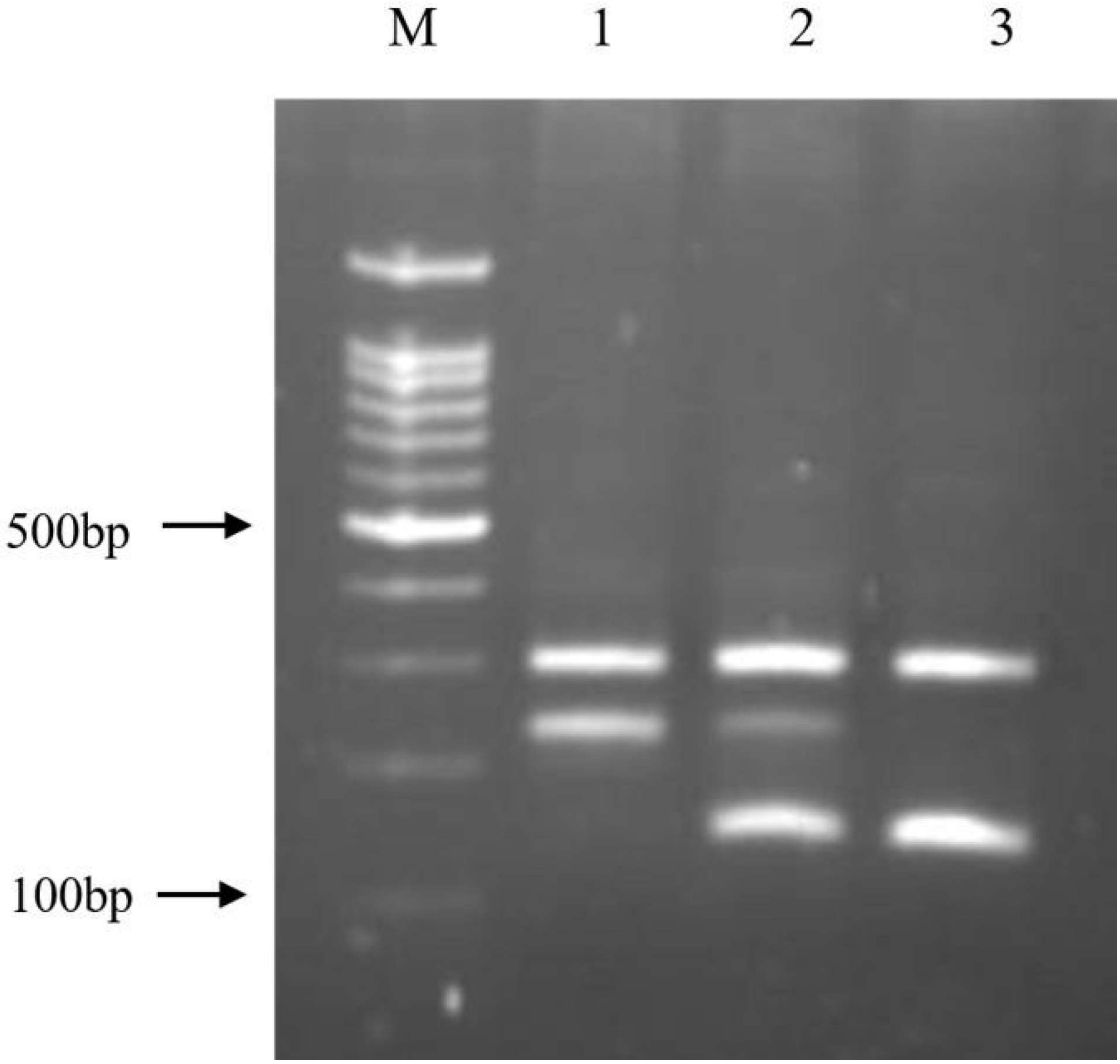

eFigure 1. Representative gel for the genotyping of *NADPH oxidase 4* (*NOX4*) rs10830278. Genotyping for *NOX4* rs10830278: Lane M, 100-bp marker; lane 1, *T/T* genotype (228-bp and 297-bp bands); lane 2, *A/T* genotype (138-bp, 228-bp and 297-bp bands); lane 3, *A/A* genotype (138-bp and 297-bp bands).

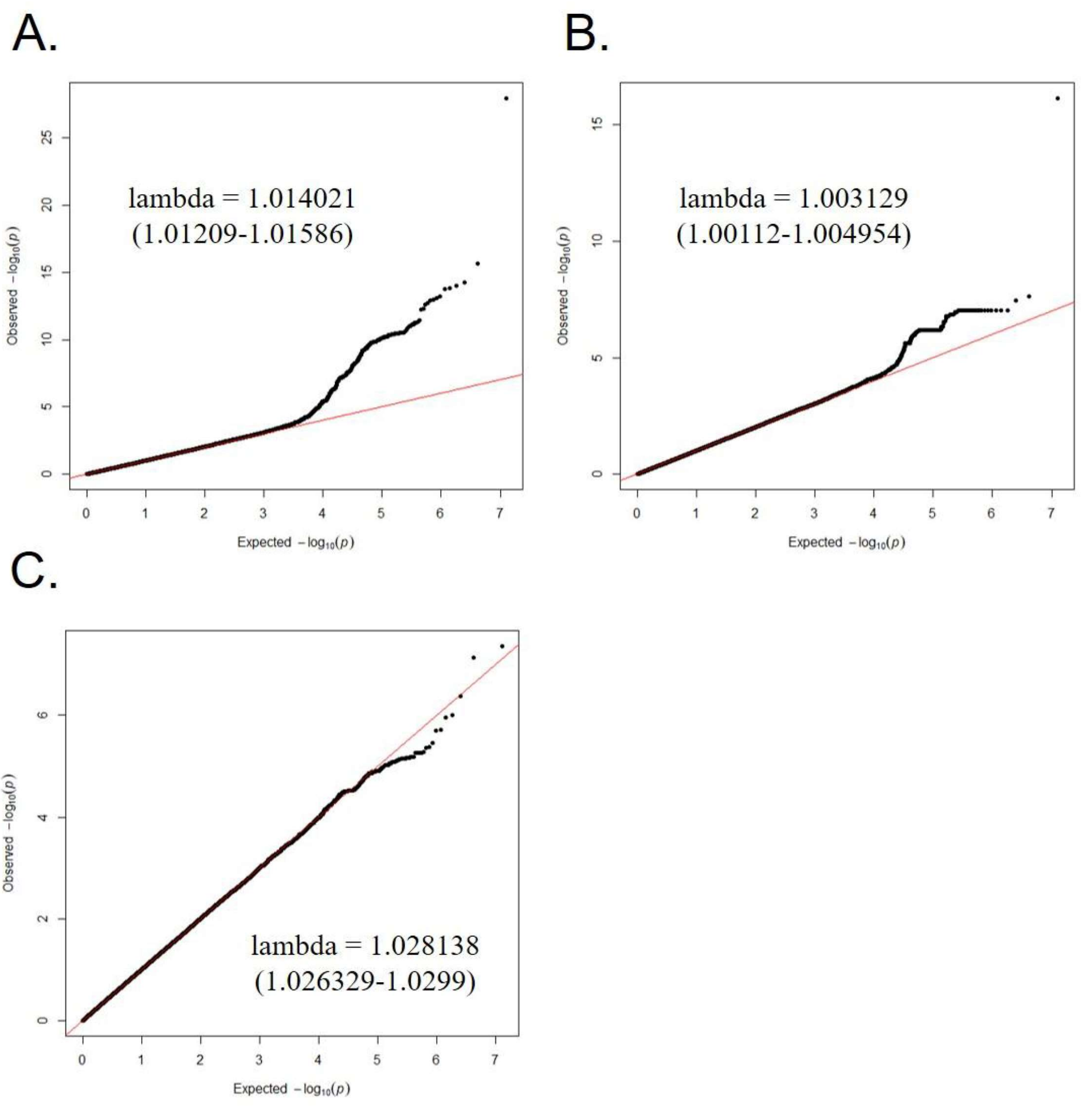

eFigure 2. Q-Q Plots for the Genome Wide Association Study (GWAS) of plasma folate metabolites of homocysteine (Hcy)/ folic acid (FA)/ vitamin B_{12} (VB_{12}) in the Japan Multi-Institutional Collaborative Cohort (J-MICC) ([**A**] Hcy: n=2,192; [**B**] FA: n=2,263; [**C**] VB_{12}: n=2,260).

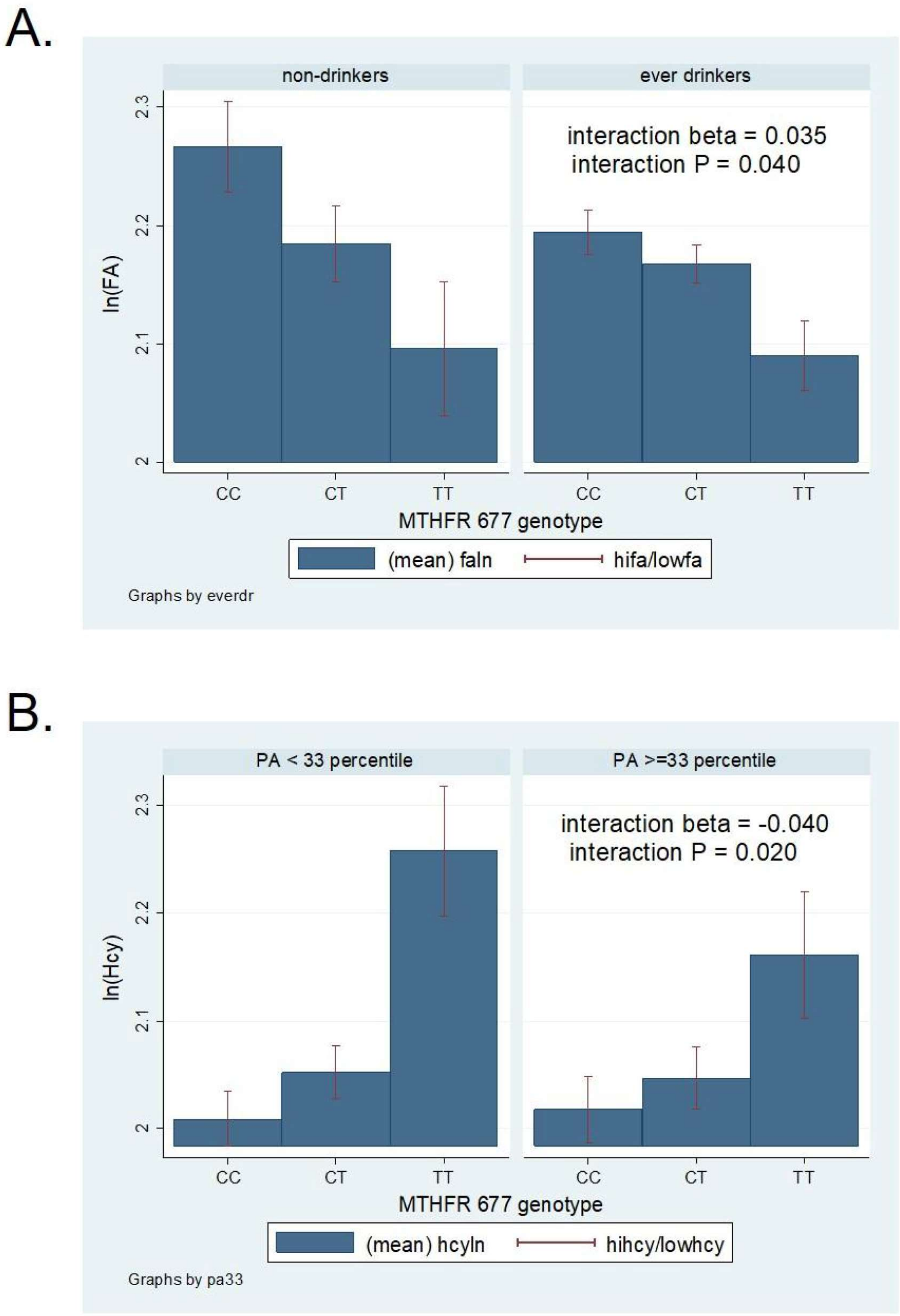

eFigure 3. Gene–environment interaction of 5-methyltetrahydrofolate reductase (*MTHFR*) C677T with lifestyle factors on plasma folate metabolites ([**A**] *MTHFR* C677T and ever drinking on folic acid [FA]; [**B**] *MTHFR* C677T and physical activity (PA) >33 percentile on Hcy).
